# Fibrolipoma, a Rare Lead Point for Adult Jejuno-Jejunal Intussusception: A Case Report

**DOI:** 10.7759/cureus.105297

**Published:** 2026-03-16

**Authors:** Rabi Farouq, Joseph E Onuh, Umar M Umar, Kevin N Ezike, Sa'ad Idris, Yidi A Mohammed

**Affiliations:** 1 Radiology, Nile University of Nigeria, Abuja, NGA; 2 Radiology, National Hospital Abuja, Abuja, NGA; 3 Nile University of Nigeria, Abuja, NGA; 4 Anatomic Pathology and Forensic Medicine, Nile University of Nigeria, Abuja, NGA; 5 Surgery, Nile University of Nigeria, Abuja, NGA

**Keywords:** adult intussusception, fibrolipoma, jejenum, jejuno-jejunal intussusception, lead point, target sign

## Abstract

Adult jejuno-jejunal intussusception is an uncommon condition usually caused by a structural lead point, often a tumor. This case describes intussusception triggered by a fibrolipoma, a benign tumor of fatty and fibrous tissue, in a patient presenting with intestinal obstruction symptoms such as abdominal pain, vomiting, and distension. Computed tomography (CT) imaging showed the classic “target sign” and identified the fibrolipoma as the lead point, which was confirmed during surgery requiring resection of both the tumor and affected jejunal segment. Although rare and often asymptomatic, fibrolipomas should be considered in adult intussusception, as timely diagnosis and surgical treatment are crucial to prevent complications like bowel ischemia.

## Introduction

Adult intussusception is an uncommon clinical entity, accounting for approximately 5% of all intussusception cases and representing only 1-5% of bowel obstructions in adults [1,2]. In stark contrast to the pediatric population, where it is often idiopathic, adult intussusception has a demonstrable lead point in over 90% of cases, necessitating thorough investigation to identify the underlying pathology [3,4]. These lead points are predominantly neoplastic, with benign lesions such as lipomas, adenomatous polyps, and gastrointestinal stromal tumors (GISTs) more common in the small bowel. In contrast, malignant lesions have a higher preponderance in colonic intussusceptions [4-6].

Lipomas of the gastrointestinal tract (GIT) are rare, benign, submucosal adipose tumors [7]. Fibrolipomas, histological variants of lipomas characterized by a prominent fibrous tissue component admixed with mature adipocytes, occur even more rarely in the GIT [8].

Computed tomography (CT) is the imaging modality of choice for evaluating suspected intussusception, providing a definitive diagnosis by revealing pathognomonic findings such as the "target sign" or a "sausage-shaped" mass [9,10]. Modern CT protocols can not only identify the intussusception but also frequently characterize the lead point, particularly if it is a lipomatous lesion exhibiting typical fat attenuation [11]. We present a case of jejuno-jejunal intussusception in a 60-year-old female caused by a fibrolipoma, discussing the clinical presentation, diagnostic challenges, and management, supported by a review of recent literature.

## Case presentation

A 60-year-old female presented with a one-year and four-month history of recurrent, severe colicky abdominal pain and dyspepsia, which was reported to worsen after meals. The symptoms were associated with occasional nausea, vomiting, and the passage of bulky stools containing undigested food particles. The abdominal pain was severe enough to deter the patient from eating. Her medical history was significant for hypertension and type 2 diabetes mellitus.

Physical examination revealed a chronically ill-looking, afebrile woman. Her vital signs were stable with a blood pressure of 119/65 mmHg and a fasting blood sugar of 4.6 mmol/L. Abdominal examination was significant for epigastric tenderness. The remainder of the physical and systemic examination was unremarkable. An extensive laboratory workup was performed. The full blood count, serum electrolytes, urea, creatinine, erythrocyte sedimentation rate (ESR), and clotting profile were all within normal limits. Investigations for tuberculosis, including Xpert® MTB/RIF assay (Cepheid, Sunnyvale, CA, USA) and acid-fast bacilli (AFB) sputum microscopy; rheumatoid factor test; and HIV screening test were all negative. The tumor marker, carbohydrate antigen (CA) 19-9, was normal at 2.11 U/mL, but the pancreatic enzyme, lipase, was mildly elevated at 106.4 U/L. Amylase level was normal at 53 U/L.

**Figure 1:**
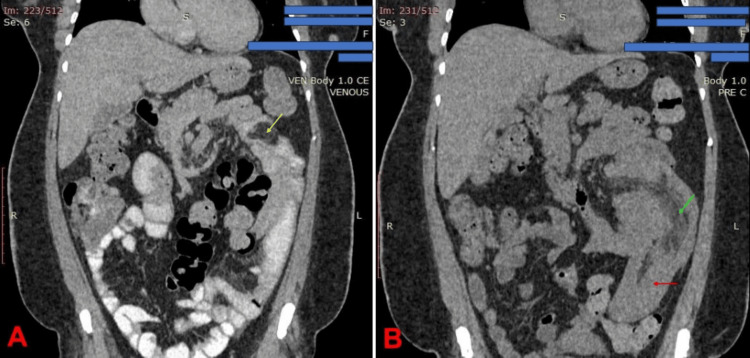
CT image of the abdomen in coronal view (A) Contrast image showing a hypodense mass appearing as a filling defect within the ileum (yellow arrow). (B) Pre-contrast image showing the mesenteric fat associated with the intussusceptum (green arrow), and the wall of the intussuscipiens (red arrow).

The diagnostic journey was protracted. A CT scan performed a year before the definitive diagnosis had shown an irregular, lobulated pancreas, leading to a suspicion of pancreatic pathology. A subsequent magnetic resonance cholangiopancreatography (MRCP) was performed, which reported a normal pancreas, biliary system, and bowel loops, and the diagnosis of intussusception was missed. Due to the persistence of symptoms, a combined contrast CT study, involving the use of oral (gastrographin) and intravenous iodinated (iohexol) contrast media, was done. This revealed a characteristic "target sign" indicative of intussusception, with a lobulated fat-density lesion identified within the bowel lumen as the lead point (Figure 1).

**Figure 2:**
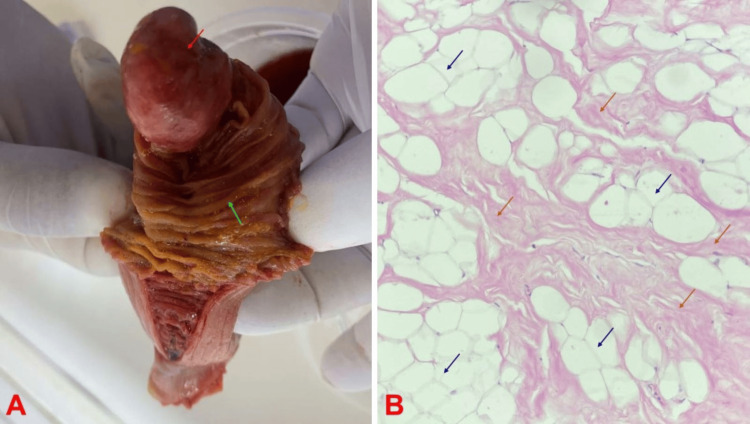
Fibrolipoma of the ileum in a 60-year-old female (A) Note pedunculated, circumscribed mass with smooth outer lining in the lumen of the ileum (red arrow) and adjacent jejunal mucosa (green arrow). (B) Note benign mesenchymal proliferation consisting of lobules of mature adipocytes (blue arrows) admixed with bands of hyalinized fibrous tissue (orange arrows)

Following the CT scan, the patient’s condition deteriorated with features of partial intestinal obstruction, and she underwent emergency exploratory laparotomy. During surgery, the jejuno-jejunal intussusception was identified as an infolding of a portion of the jejunum into a distal jejunal segment and manually reduced, with the lead point being a pedunculated mass in the jejunum, approximately 6cm in its widest dimension, located about 90cm from the duodenal junction. A jejunal segment, incorporating the mass, was resected, and a primary jejuno-jejunal anastomosis was performed. The resected specimen was sent for histopathological analysis, which confirmed the diagnosis of a fibrolipoma (Figure 2).

The patient’s post-operative condition was satisfactory, and she was discharged home on the seventh day after the removal of stitches. She remained symptom-free on her two-week and one-month follow-up visits.

## Discussion

This case exemplifies the diagnostic challenges associated with adult intussusception, particularly when it is intermittent in nature. The initial misdirection towards pancreatic pathology, based on a nonspecific CT finding and a mildly elevated lipase, delayed the correct diagnosis for over a year. This underscores the importance of considering intussusception in the differential diagnosis for adults presenting with recurrent, obstructive abdominal symptoms, even when initial investigations are inconclusive [12].

The definitive role of cross-sectional imaging, specifically CT, is clearly demonstrated here. While the initial CT and subsequent MRCP failed to identify the problem, due to the intermittent nature, the combined contrast CT study was pivotal in visualizing the pathognomonic signs of intussusception and characterizing the lead point [13]. The identification of a well-circumscribed, fat-attenuation lesion within the intussusceptum is highly suggestive of a lipoma or its variants, such as a fibrolipoma [10,11]. This pre-operative characterization is invaluable for surgical planning, as it can raise suspicion for a benign lesion, although malignancy must always be ruled out [4,6].

Fibrolipomas are a rare histological subtype of gastrointestinal lipomas [8]. While simple lipomas are the most common benign, mesenchymal tumors of the GIT, those with a significant fibrous component are less frequently reported [7,14]. Their potential to cause intussusception arises from their size and mobility, particularly if they are pedunculated. As they are pliable, they may lead to intermittent, self-reducing intussusception, which explains the chronic, relapsing symptoms in our patient [8,9].

The management of adult intussusception is invariably surgical, given the high likelihood of a pathological lead point and the risk of complications like ischemia, perforation, or necrosis [3,15]. The surgical approach, whether reduction before resection or en bloc resection without reduction, remains a subject of debate and is influenced by the location of the intussusception and suspicion of malignancy [5,15,16]. In this case, the surgeons opted for reduction followed by a limited resection and primary anastomosis, an approach often favored in small bowel intussusceptions where the lead point is pre-operatively suspected to be benign [6,17]. The final diagnosis rests on histopathological examination, which confirmed the benign nature of the lesion and validated the surgical strategy [18].

## Conclusions

This case of adult jejuno-jejunal intussusception secondary to a fibrolipoma underscores the diagnostic challenges inherent in this rare condition. It highlights the necessity of maintaining a high index of suspicion for intussusception in adults presenting with recurrent, obstructive abdominal symptoms, even in the context of a protracted and intermittently symptomatic clinical course. Computed tomography is affirmed as the cornerstone of diagnosis, proving capable of not only identifying the intussusception but also of characterizing the nature of the lead point, thereby critically informing surgical planning. While uncommon, a fibrolipoma should be considered in the differential diagnosis of benign lead points. Ultimately, this case reinforces that a successful outcome hinges on a multidisciplinary approach involving radiologists, surgeons, and pathologists to ensure timely diagnosis, appropriate intervention, and the prevention of serious complications such as bowel ischemia.
